# Application of a patient-derived xenograft model in cytolytic viral activation therapy for nasopharyngeal carcinoma

**DOI:** 10.18632/oncotarget.5544

**Published:** 2015-09-24

**Authors:** Cheng-Lung Hsu, Yung-Chia Kuo, Yenlin Huang, Yin-Cheng Huang, Kar-Wai Lui, Kai-Ping Chang, Tung-Liang Lin, Hsien-Chi Fan, An-Chi Lin, Chia-Hsun Hsieh, Li-Yu Lee, Hung-Ming Wang, Hsin-Pai Li, Yu-Sun Chang

**Affiliations:** ^1^ Division of Hematology-Oncology, Department of Internal Medicine, Chang Gung Memorial Hospital, Chang Gung University, Taoyuan 333, Taiwan, ROC; ^2^ Department of Pathology, Chang Gung Memorial Hospital, Chang Gung University, Taoyuan 333, Taiwan, ROC; ^3^ Division of Neurologic Surgery, Department of Surgery, Chang Gung Memorial Hospital, Chang Gung University, Taoyuan 333, Taiwan, ROC; ^4^ Department of Medical Imaging and Intervention, Chang Gung Memorial Hospital, Chang Gung University, Taoyuan 333, Taiwan, ROC; ^5^ Department of Otolaryngology-Head and Neck Surgery, Chang Gung Memorial Hospital, Chang Gung University, Taoyuan 333, Taiwan, ROC; ^6^ Department of Cell and Molecular Biology, Chang Gung University, Taoyuan 333, Taiwan, ROC

**Keywords:** NPC, PDX, EBV, target therapy, viral lytic therapy

## Abstract

Nasopharyngeal carcinoma (NPC) is an Epstein Barr virus (EBV)-related malignancy in which the tumor microenvironment plays a pivotal role in tumor progression. Here, we developed two patient-derived xenograft (PDX) mouse lines from engrafted NPC metastatic tumors. Positive staining for EBV-encoded small RNAs confirmed that these tumors harbored EBV, and gene expression profile analyses further showed that the PDX was highly similar to the primary parent tumor. *In vivo* drug screening using the PDX system demonstrated that gemcitabine had the best antitumor effect among the tested drugs. The donor of this PDX also showed excellent responsiveness to gemcitabine treatment. The combination of gemcitabine and valproic acid exerted synergistic antitumor effects. Further addition of ganciclovir to this two-drug combination regimen enhanced cytolytic viral activation, yielding the best antitumor response among tested regimens. Treatment with this three-drug combination regimen decreased plasma EBV-DNA load, tumor viral concentration, and the number of viable tumor cells to a greater extent than the two-drug gemcitabine and valproic acid combination. These results highlight the value of PDX models in the development of EBV-targeted strategies to treat NPC.

## INTRODUCTION

Nasopharyngeal carcinoma (NPC) is highly prevalent in Southern Chinese populations and has a predilection to affect young adult males [1]. In this endemic region, non-keratinizing and undifferentiated carcinoma constitutes up to 99% of all cases, and these tumors are closely related to infection with Epstein Barr virus (EBV) [2, 3]. The presence of EBV in virtually all tumor cells highlights the integral relationship among virus, cancer cells, and tumor microenvironment [3]. In the past decade, the amount of free EBV-DNA in peripheral blood cells has been shown to correlate well with tumor stage, and has essentially become a standard marker for NPC [4]. EBV-DNA load has been identified as an independent prognostic factor in metastatic NPC patients such that those with a high pretreatment viral load have worse outcomes [5–7].

There has been a recent increase in the use of patient-derived xenografts (PDX) as a preclinical model [8]. In application, the tumor from a patient is directly implanted into a severe combined immunodeficiency (SCID) mouse and then re-implanted into subsequent passage mice to amplify the tumor mass. Several PDX models have been established. These models exhibit a stable biological profile when passaged in mice (especially during early passages) in terms of global gene-expression patterns, mutational status, metastatic potential, drug responsiveness, and tumor architecture [8]. Comprehensive genome-wide gene expression analyses have demonstrated that early passage PDX have key genomic expression profile features similar to those of primary tumors [9]. In head and neck cancer, the PDX model had been shown to harbor mutations in *TP53* or exhibit amplification of *CCND1* similar those observed clinically in association with cisplatin resistance [10, 11]. Moreover, because orthotopic PDX models preserve a greater proportion of stromal components, they recapitulate tumor microenvironment effects, developing patterns of locoregional and distant metastases similar to those of human tumors [12, 13]. Primary tumor xenografts have been shown to be valuable in a tailored personalized medicine setting for drug-sensitivity screening in cases where standard treatment has failed; they could also help to identify key pathway components suitable for targeted drug development [14].

The PDX model had been applied in NPC to study combinations of chemotherapy, radiotherapy and histone deacetylase (HDAC) inhibitors [15], and for characterizing the role of the microRNA, *miR-31,* in EBV-associated NPC [16].

In a cancer cell, EBV is in a latent phase and expresses 8–11 genes involved in maintaining EBV proliferation. Among the latent genes expressed in NPC, latent membrane protein 1 is considered the primary viral oncoprotein, facilitating tumor cell growth and local invasion, and conferring antiapoptotic properties and survival advantages [17]. EBV reactivation from latency requires expression of viral immediate-early transactivators of subsequent lytic genes, including thymidine kinase, protein kinase, and an EBV-encoded DNA polymerase. These gene products are essential for creating new viral genomes [18]. This EBV latent-lytic shift may offer a strategy for promoting EBV-dependent tumor cell killing. Some therapeutic agents, such as the HDAC inhibitor bortezomib, a variety of chemotherapy agents and irradiation, have been shown to induce the ATM-p53 pathway, which would promote EBV reactivation [19–23]. In lytic-induction strategies, some EBV-encoded kinases may convert nucleoside analogs such as ganciclovir into cytotoxic drugs that kill EBV-positive tumor cells and virus [20, 24]. Furthermore, the phosphorylated form of ganciclovir can be transferred to adjacent tumor cells through gap junctions, resulting in “bystander killing” of a much greater percentage of tumor cells [20].

## RESULTS

### Establishment and characterization of the EBV-positive PDX model

**Figure 1:**
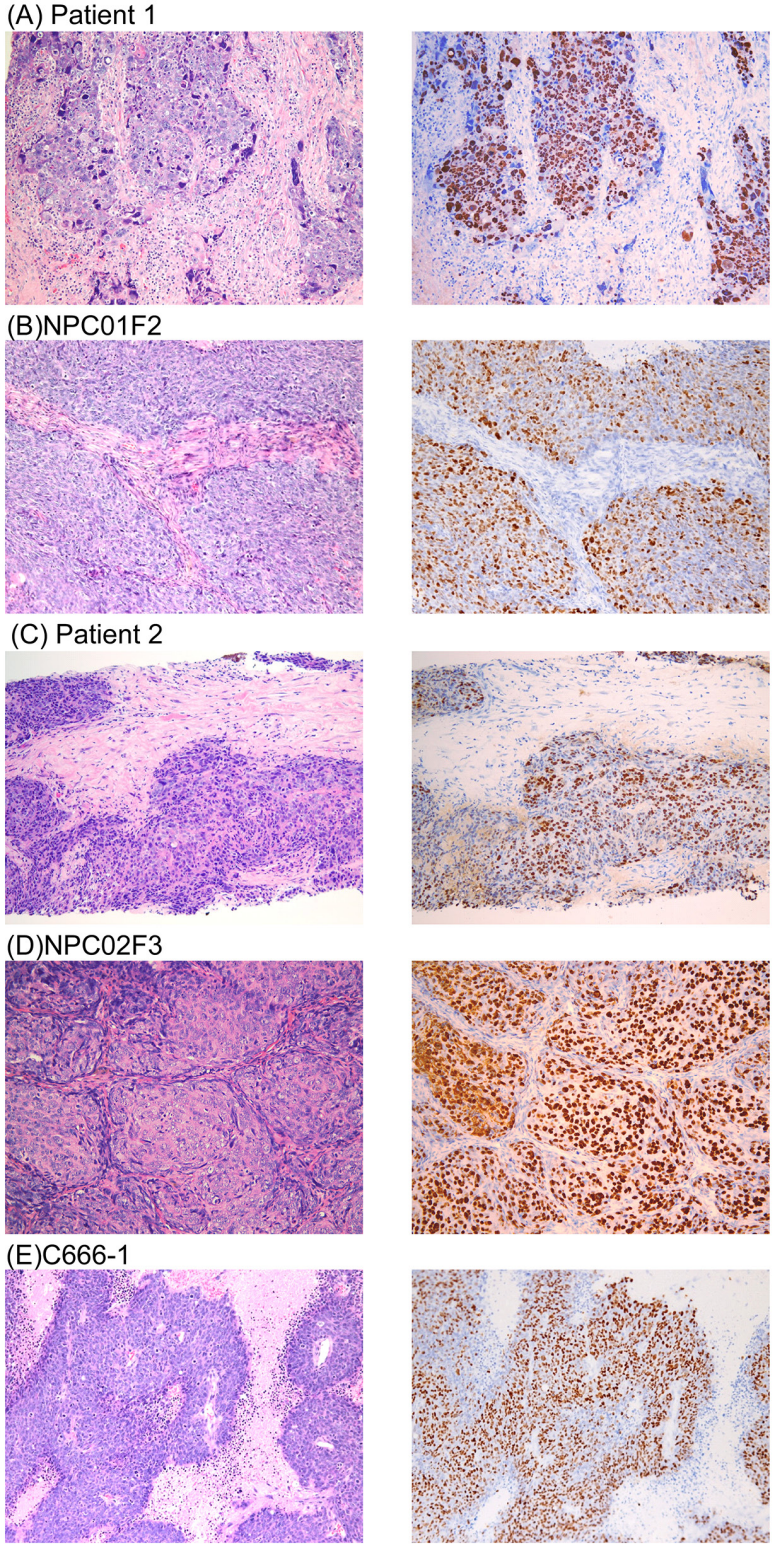
Histological comparison of the parent clinical tumor **A, C.** with PDX **B, D.** and NPC cell line (C666-1) xenografts **E.** H&E staining (left column) and detection of EBER by *in situ* hybridization (right column). Original magnification, 200X.

Very few currently available NPC cell lines harbor endogenous EBV, which is important for NPC cell growth and progression. Furthermore, microenvironment-cancer cell interactions play a pivotal role in cancer cell progression in NPC. To address these issues, we sought to establish EBV-positive tumors via PDX in a SCID mouse model. Exploiting the fact that engrafting rate is higher for metastatic tumors than primary site tumors, we established two mouse xenograft lines from NPC metastatic tumors: NPC01, obtained from a paraspinal soft tissue tumor at initial diagnosis (before treatment) of NPC with bone and soft tissue metastasis, and NPC02, obtained from a neck lymph node biopsy after three lines of chemotherapy. Compared with the EBV-positive NPC cell line C666-1, a xenograft with a homogeneous single tumor cell type, PDX had a more heterogeneous histologic phenotype with various size tumor cells and background cells (Fig. 1). EBER staining confirmed that these tumors harbored EBV (Fig. 1). The PDX required a longer time to grow (3–6 months for one passage) than the cell line xenograft (~2–3 weeks from implantation of 5 × 10^5^ cells to achieving a 1,000 mm^3^ xenograft).

**Figure 2:**
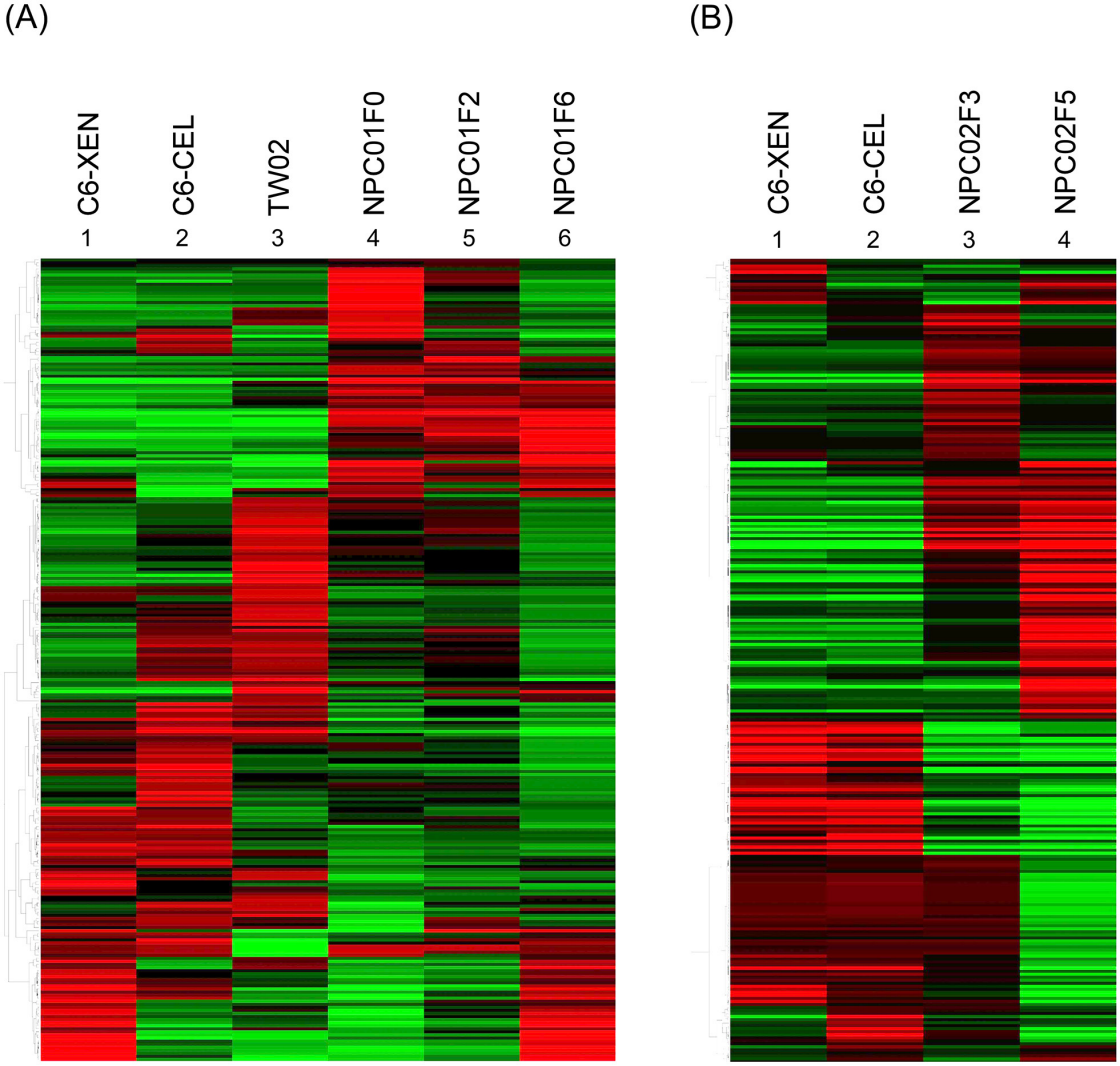
Comparison of gene expression profiles between clinical samples and PDX by microarray analysis **A.** NPC01 was from a paraspinal soft tissue tumor obtained at initial diagnosis (before treatment) of NPC with bone and soft tissue metastasis. **B.** NPC02 was obtained from a small piece of neck lymph node biopsy after three lines chemotherapy; it could be amplified in the NOD/SCID mice system, but a parent clinical sample was unavailable for microarray analysis. Abbreviations: C6-XEN, C666-1 xenograft; C6-CEL, C666-1 dish culture; TW02, NPC cell line, dish culture; NPC01F0, NPC patient 1 excision metastatic tumor; NPC01F2/NPC01F6, second and sixth passage PDX of NPC01F0; NPC02F3/NPC02F5, third and fifth passage PDX of NPC patient 2 neck lymph node biopsy.

An initial small biopsy/excisional tumor was amplified in the PDX system. An mRNA microarray analysis of these tumors compared with *in vitro*-cultured NPC cell lines and a C666-1 xenograft showed that the gene profile of the PDX tumor was more similar to the clinical sample than the C666-1 xenograft and was clearly different from that of *in vitro*-cultured EBV-positive and -negative cell lines (Fig. 2A). Expression levels of some hematopoietic/macrophage-related genes were lower in the PDX than in the parent tumor, possibly reflecting the immunocompromised background of SCID mice. Because initial biopsy tissue pieces were small, we lacked sufficient parent human tumor for gene expression profile analysis of NPC02; however, the gene expression profile of this PDX line was still different from that of the C666-1 xenograft (Fig. 2B). Collectively, these results confirm that the PDX system reliably represents the clinical sample.

### Drug screening using an *in vitro* NPC cell line versus the *in vivo* PDX system

**Figure 3:**
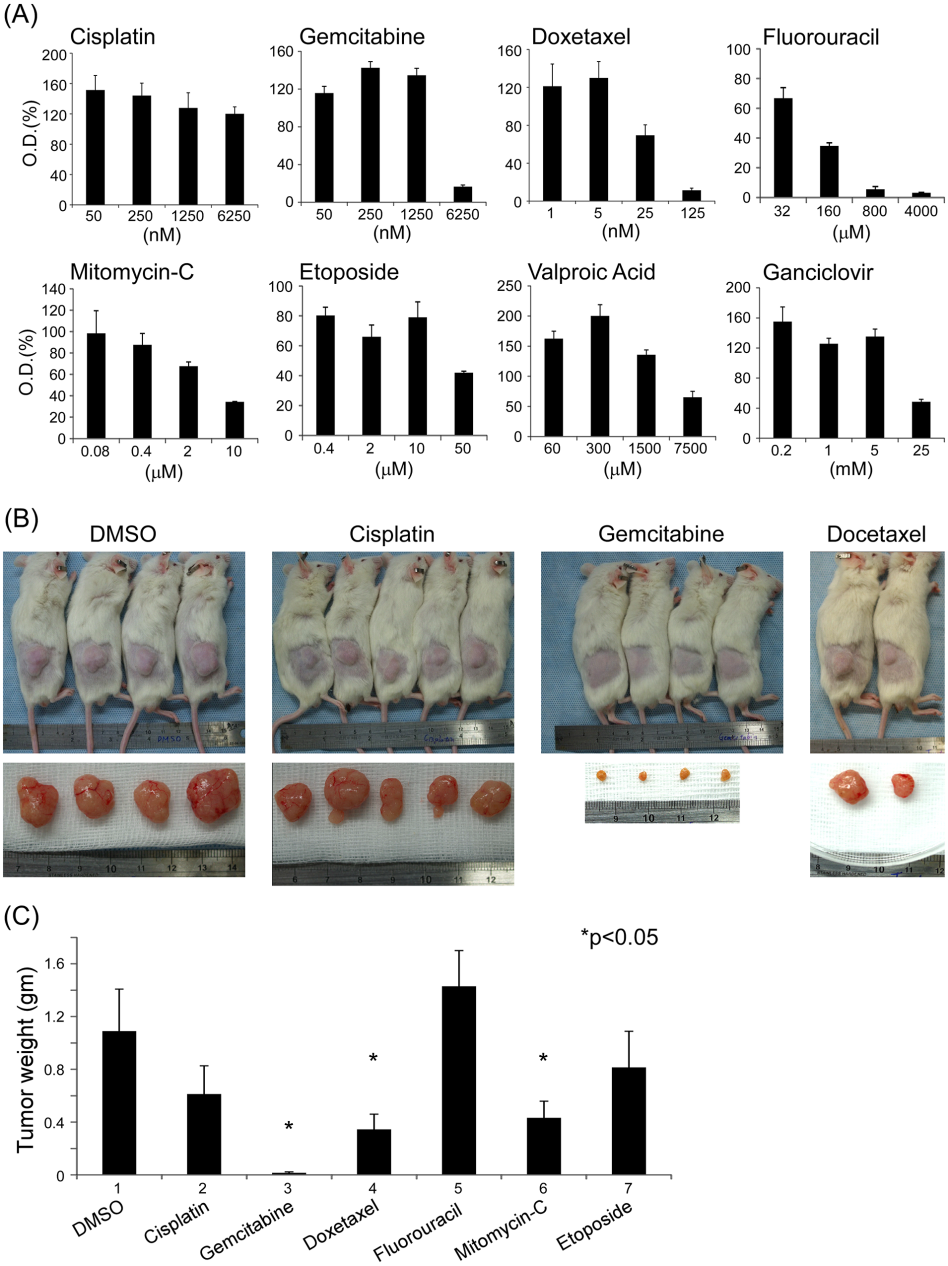
Drug-sensitivity screening *in vitro* using NPC C666-1 cell line growth assays A. and *in vivo* using the PDX model (B, C) **A.** C666-1 cells were plated at 5 × 10^4^ cells/well in 24-well plates and incubated with or without different concentrations of tested drugs for 6 days. Cell growth was assessed using the MTT assay. Values presented in figures are mean OD_590_ ± SD from at least three independent reaction wells. **B.** NOD/SCID PDX subcutaneous tumor before and after excision. **C.** Xenograft tumor weight after sacrificing mice. After the tumor had been sub-implanted in NOD/SCID mice and the xenografts had reached a volume of approximately 150 mm^3^, animals were randomized (3–5 tumor-bearing mice per group) and various drug dose schedules, described in Materials and Methods, were administered via intraperitoneal injection. Mice were sacrificed 3 months after chemical injection or earlier in circumstances involving declining health status, morbundity, or unrelieved pain and discomfort. Tumor weights presented in figures are means ± SD from at least three independent studies. The PDX used in these studies were from the first eight passages of each line.

After establishing this reliable tumor system, we used it to test sensitivities to the most commonly clinically used anticancer chemicals, using activity in *in vitro* cultured C666-1 cell line as a guide. Docetaxel, gemcitabine, and mitomycin-C showed good anticancer activity in the nano- to micromolar concentration range, although the IC_50_ for docetaxel was lower than that for gemcitabine and mitomycin-C (Fig. 3A). In contrast, cisplatin, fluorouracil, etoposide, and valproic acid were largely ineffective except at higher concentrations (Fig. 3A). Notably, millimolar concentrations of valproic acid and ganciclovir were required to produce antitumor effects. We next tested these drugs in the PDX system. To our surprise, gemcitabine had the best anticancer effect among the tested drugs in the PDX model (Fig. 3B and 3C). Although cisplatin is effective in clinical practice, it was ineffective at the doses tested in the PDX system. Some diarrhea episodes were noted in the docetaxel treatment group. Mitomycin-C was also found to be an active drug in this *in vivo* assay (Fig. 3C).

### Drug sensitivity in PDX assays correlates with patient clinical response

**Figure 4:**
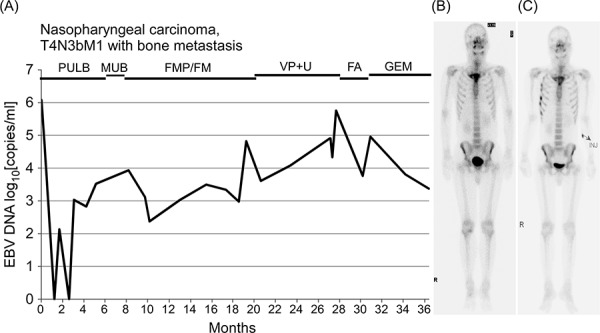
PDX drug sensitivity correlates with clinical response NPC02, a 42 year-old NPC patient with bone metastasis, had received five different lines of palliative chemotherapy before gemcitabine (GEM) was prescribed. Allergy to cisplatin was found during treatment with the third chemotherapy regimen (FMP); thus, cisplatin was subsequently omitted. **A.** Plasma EBV-DNA load reflecting the clinical response of the patient. Plasma EBV-DNA load decreased after treatment with GEM, which was also shown to be effective in the corresponding NPC02 PDX system. The patient died from aspiration pneumonia with septic shock 3 months after the last plasma EBV-DNA detection. Whole-body bone scan before **B.** and after **C.** GEM treatment at 3-month intervals, revealing stable disease (hot spots in right chest wall ribs in (C) are due to trauma). Abbreviations: PULB, cisplatin, tegafur/uracil, leucovorin, and bleomycin; MUB, methotrexate, tegafur/uracil, and bleomycin; FMP, tegafur/uracil, mitomycin-C, and cisplatin; FM, tegafur/uracil and mitomycin-C; VP+U, etoposide and tegafur/uracil; FA, tegafur/uracil and doxorubicin.

Patient number one died due to cancer progression before we were able to finish his drug-sensitivity screening in the PDX system. Gemcitabine had been shown to exert the best anticancer effect in our *in vivo* NPC02 PDX system. Accordingly, we tested gemcitabine in patient number two, who had been treated unsuccessfully with five different anticancer treatments (Fig. 4A). Follow-up clinical assessments showed a decreased plasma EBV-DNA load (Fig. 4A) and diminished toxicity profile (not shown) in response to gemcitabine treatment, and an evaluation of drug response by bone scan showed stable disease (Fig. 4B and 4C).

### Application of the PDX model to cytolytic viral activation therapy

**Figure 5:**
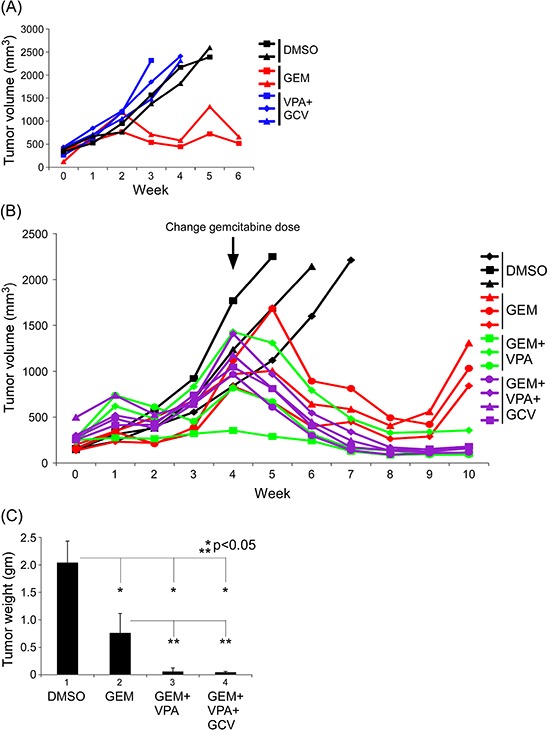
Tumor suppressive effects of different gemcitabine-based regimens in the PDX model **A.** Valproic acid (VPA) combined with ganciclovir (GCV) had no antitumor effect in this PDX model. The effect of gemcitabine (GEM) alone (2 mg/kg) is shown for comparison. After the NPC02F3 PDX had been sub-implanted in NOD/SCID mice and the xenograft had reached a volume of approximately 1000 mm^3^, animals were randomized (2–3 tumor-bearing mice per group) and treated with the following dose schedule: DMSO 100 μl, 5 times/wk; GEM, 2 mg/kg, 5 times/wk; VPA, 50 mg/kg, 5 times/wk; and GCV, 50 mg/kg, 5 times/wk. All chemicals were applied for 2 weeks every 3 weeks. **B.** Effects of GEM-based regimens in the PDX system. Low-dose GEM (1 mg/kg) given during the first 4 weeks of treatment showed little or no antitumor effect in GEM-containing groups. Starting on week 5, the GEM dose was increased to 2 mg/kg. After sub-implanted NPC02F5 PDX had reached a volume of approximately 500 mm^3^, animals were randomized (3–4 tumor-bearing mice per group) and treated with the following dose schedule: DMSO 100 μl, 5 times/wk, 1 wk/3 wk; GEM, 1 mg/kg, 5 times/wk, 1 wk/3 wk; VPA, 50 mg/kg, 5 times/wk, 1 wk/3 wk for GEM + VPA group and 2 wk/3 wk for GEM + VPA + GCV group; and GCV, 50 mg/kg, 5 times/wk, 2^nd^ wk/3 wk. At the beginning of the fifth week, all treatment schedules were reset and the same dose schedule was applied except the GEM dose was increased to 2 mg/kg. **C.** Xenograft weights for different treatment regimens in (B)

Cytolytic viral activation therapy (CVAT), which shifts oncolytic viruses from the latent phase to the lytic phase and causes cancer cell death, has recently come to be considered a therapeutic strategy in EBV-related cancers, including lymphoma and NPC [25, 26]. Different combinations of standard chemotherapeutic agents, HDAC inhibitors, and antiviral agents have been reported to exert efficient anticancer activity toward lymphoma and NPC [19, 21, 22, 26]. In initial tests of this concept in our PDX system, we found that, whereas gemcitabine alone effectively suppressed tumor growth, the combination of valproic acid and ganciclovir had no significant antitumor effect compared with DMSO controls (Fig. 5A, valproic acid + ganciclovir vs. gemcitabine). We confirmed the importance of the standard chemotherapeutic agent, gemcitabine, in these combination regimens by using a reduced dose of gemcitabine during the first 4 weeks of treatment, which produced little or no antitumor effect (Fig. 5B). A combination regimen consisting of gemcitabine and valproic acid exerted superior antitumor effects compared with gemcitabine alone (Fig. 5B, gemcitabine + valproic acid vs. gemcitabine). Further addition of ganciclovir to this gemcitabine + valproic acid regimen produced tumor control similar to that observed in the gemcitabine + valproic acid group (Fig. 5B, gemcitabine + valproic acid + ganciclovir vs. gemcitabine + valproic acid). Xenograft weights measured after sacrificing mice confirmed these tendencies (Fig. 5C).

**Figure 6:**
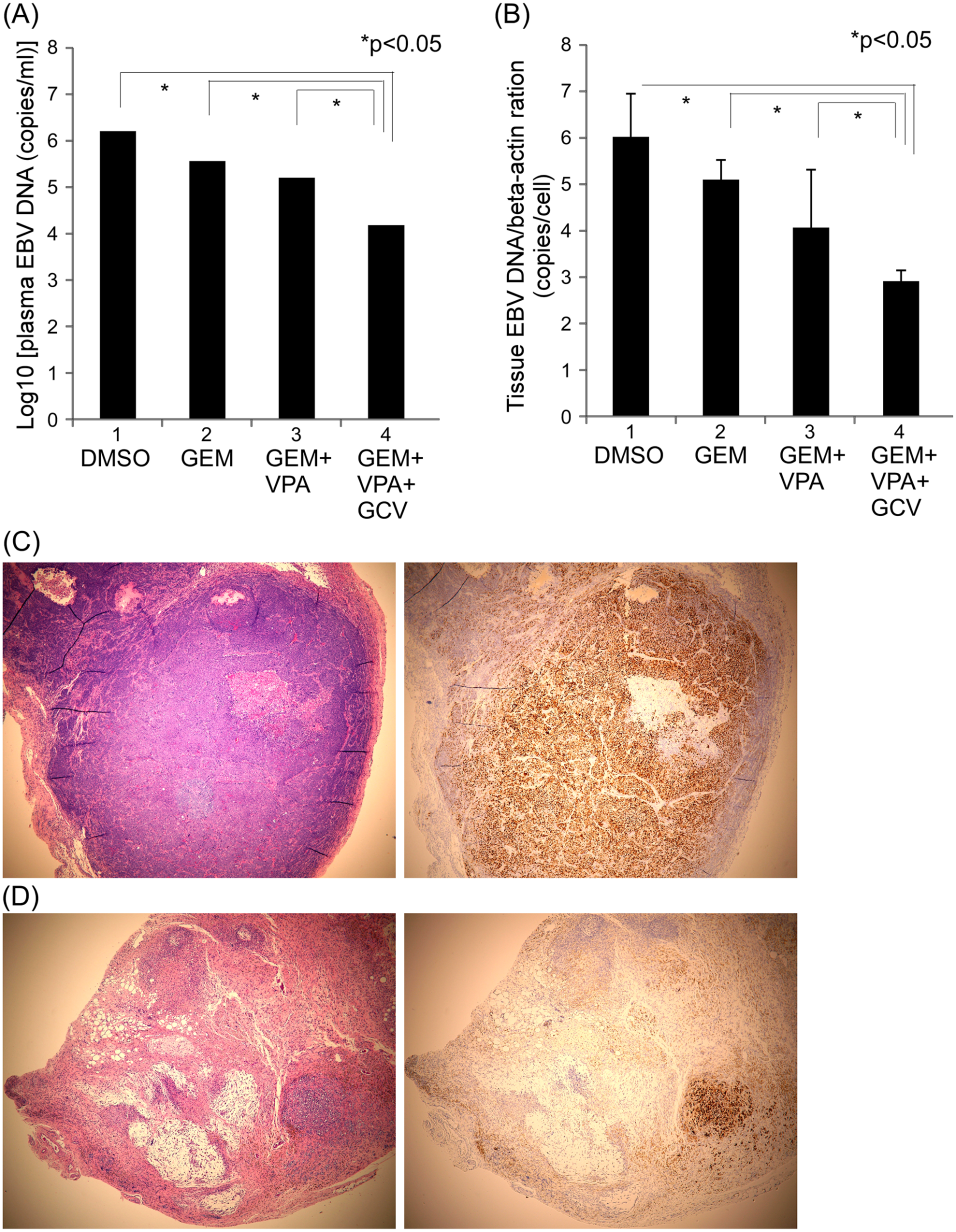
EBV activity decreased in the ganciclovir-containing treatment group **A.** Plasma EBV-DNA load and **B.** xenograft tissue viral concentration. NPC02F6 PDX were used in these studies with two cycles of the same drug schedule as shown in Figure 5B. Tumor sections from the ganciclovir (GEV) + valproic acid (VPA) treatment group **C.** and gemcitabine (GEM) + VPA + GCV treatment group **D.** *Left*: H&E staining; *right*: EBER staining. Original magnification, 40x.

Although adding ganciclovir had no additional tumor-controlling effect, it is possible that it affected EBV-related activity. In support of this, we found that the ganciclovir-containing three-drug combination group not only had a lower plasma EBV-DNA load, but also a lower EBV-DNA/tumor cell ratio in tumor tissue than the two-drug gemcitabine + valproic acid group (Fig. 6A and 6B). Furthermore, a histological examination of hematoxylin and eosin (H&E)-stained tumors after treatment showed fewer viable tumor cells in the ganciclovir-containing three-drug group than in the gemcitabine + valproic acid group. EBER *in situ* hybridization staining also showed fewer EBER-positive cells in the three-drug treatment group (Fig. 6C and 6D). These results suggest that the ganciclovir-containing three-drug regimen is the best combination among those tested.

## DISCUSSION

NPC is an EBV-related cancer in which the tumor microenvironment has a crucial role in tumor progression. Here, using metastatic tumors, which have greater engrafting potential than primary site tumors in the PDX model [27], we established EBV-positive PDX lines from two patients with metastatic NPC, including one from a neck lymph node core biopsy sample. Maintaining these xenograft lines may help retain EBV in tumor cells, and subsequently transferring these xenografts to an *in vitro* culture system could enable the establishment of new EBV-positive NPC cell lines.

Our microarray analyses showed that the gene expression profile of the PDX tumor was highly similar to that of the parent tumor, confirming that the PDX system recapitulates features of the clinical sample. In our PDX drug-sensitivity screens, gemcitabine was the best candidate among the commonly used therapeutic agents tested. We further clinically validated drug sensitivity results obtained with cell line/PDX models in the original patient (Fig. 4), showing that gemcitabine exerted excellent anticancer effects in the corresponding patient, who had been heavily and unsuccessfully treated with a number of chemotherapeutic regimens. We also demonstrated that gemcitabine plays a decisive role in combination CVAT regimens (Fig. 5B). Although docetaxel was found to be superior to gemcitabine in our *in vitro* cell line-based drug-sensitivity screens, it showed limited efficacy in the PDX system owing to toxicity, which manifested as diarrhea. Mitomycin-C was also active in PDX screens and this activity was correlated with a positive clinical response to mitomycin-C, used as a third-line treatment, that was maintained for almost 1 year. The combination of HDAC-inhibitor (valproic acid) and antiviral agent (ganciclovir), which has shown some efficacy in certain lymphoma models [28], was not effective in our NPC PDX model (Fig. 5A).

The anticancer drugs tested demonstrate efficacy in clinical practice, but their response rates are variable—generally less than 50% [29]. The high tumor heterogeneity within and between tumors in a given patient and between tumors in different patients is likely a contributing factor to this variability. Notable in this context, the current PDX was derived from tumors from only two cases amplified in NOD/SCID mice. Taken together, these observations may help explain why some commonly used chemotherapeutic agents were ineffective in our PDX system.

The PDX system has previously been applied for drug-sensitivity screening and target-gene identification [8, 30, 31]. The true value of the PDX model for drug sensitivity testing lies in its role as a bridge between cell line studies and clinical tests, but it still has limitations, including restrictions associated with surgical/biopsy tissue processing, technically complicated orthotopic implantation, limited availability of original source material, low take out rate, and the time-consuming nature of xenograft growth [32, 33]. Different animal species also have different drug sensitivities and/or tolerances; for example, docetaxel has greater gastroenteritis side effects in NOD/SCID mice than in humans. Another potential weak point is the immunocompromised background of mice, which could limit its utility, since some inflammation/immune-related phenomena may be abolished in this system [8]. We also observed that some hematopoietic/macrophage-related genes were expressed at a low level in a PDX background. A high percentage of lymphoma (32.5%) in PDX attributable the lymphomagenesis background of NOD/SCID mice has also recently been reported [34]. In this context, approximately 5% of our PDX-positive mice showed contamination by this spontaneous lymphomagenesis, with rapid progression of the tumor after passage.

EBV is considered a therapeutic target in EBV-related cancers, including lymphoma and NPC [25]. Valproic acid, a short chain fatty acid used clinically as an anti-epilepsy drug, could have some HDAC-inhibitory effects [35]. This HDAC inhibition may sensitize cancer cells to the effects of gemcitabine, and the combination of the drugs in these two categories has been shown to exert synergistic anticancer effects [22]. Our current PDX model, which revealed the key role of gemcitabine in combination regimens, also confirmed this concept. More recently, CVAT composed of a standard chemotherapeutic agent (gemcitabine), an HDAC inhibitor (valproic acid), and an antiviral agent (ganciclovir) has shown promising anticancer effects in refractory NPC patients [26]. Although the similar tumor control achieved with this three-drug combination regimen and the two-drug gemcitabine + valproic acid combination would seem to raise questions about the role of ganciclovir (Fig. 5), a detailed analysis demonstrated diminished EBV-related activity in the ganciclovir-containing, three-drug treatment group, as evidenced by lower plasma EBV-DNA load, decreased tumor EBV concentration, and fewer viable EBV-positive tumor cells (Fig. 6). These results suggest that the ganciclovir-containing three-drug regimen is the best combination among those tested. Although chemotherapy-related toxicity observed in patients treated with this ganciclovir-containing three-drug regimen is reported to be mild [26], additional clinical trials will be required to further confirm the reduced side effects, as well as the excellent tumor control efficacy, of this three-drug combination CVAT.

## MATERIALS AND METHODS

### Materials

Cisplatin, gemcitabine, docetaxel, etoposide, 5-fluorouracil (fluorouracil), mitomycin-C, valproic acid, and ganciclovir were purchased from Sigma Chemical Co (St. Louis, MO).

### Cell growth assay

C666-1 cells were grown in RPMI medium containing 10% fetal bovine serum (FBS). Cells were plated at 5 × 10^4^ cells/well in 24-well plates and incubated with or without different concentration of tested drugs for 6 days. Cell growth was assessed using the MTT (3-{4,5-dimethylthiazol-2-yl}-2, 5-diphenyltetrazolium bromide) assay [36]. At the end of studies, 50 μl of a 5-mg/mL MTT solution was added to each well containing 500 μl of medium, and plates were incubated for 3 hours; 500 μl of isopropyl alcohol was then added to dissolve the reduced formazan product. The absorbance of each well was measured at a wavelength of 590 nm in a DU 640B spectrophotometer (Beckman, Fullerton, CA) according to the manufacturer's protocol. Values presented in figures are mean OD_590_ ± SD from at least three independent reaction wells.

### Animal studies

All experiments involving laboratory animals were done in accordance with the Guideline for Animal Experiments of Chang Gung Memorial Hospital and were approved by the Animal Research Committee at Chang Gung Memorial Hospital.

The EBV-expressing NPC C666-1 cell line was used as a positive control. The cancer cells were harvested, washed twice with phosphate-buffed saline (PBS), and resuspended at a final concentration of 5 × 10^6^ cells/mL in Matrigel (BD Biosciences, San Jose, CA) containing basement membrane components. Then, 5 × 10^5^ cells (100 μL per site) were subcutaneously injected into the flanks of 4-6-week-old male NOD/SCID mice (BioLASCO, Taiwan). Tumor development was confirmed within 2–3 weeks after injection of the same number of cell sub-clones. Treatment with different chemicals was initiated at the same time. Tumor dimensions were measured twice a week with calipers, and tumor volume was calculated with the formula, tumor volume (mm^3^) = tumor length (mm) × [tumor width (mm)]^2^ × 0.5. Tumors were harvested for further analysis. Three to five mice for each group (with or without chemical treatment) were used. Mice were sacrificed 3 months after chemical injection or earlier if tumors reached a size greater than 2000 mm^3^, body weight loss exceeded 20%, mice were unable to maintain their normal food and water intake for 3 days, had micturition or defecation difficulties, or other conditions that would violate humane treatment regulations.

### Statistical analysis

Cell line and tumor weight studies data are presented as means ± SD. Final tumor volumes were compared using a two-tailed analysis of variance (ANOVA).

### Patient enrollment

Two biopsy-proven NPC patients with local recurrence or distant metastasis were enrolled between July 2013 and June 2014. Written informed consent approved by the Institutional Review Board of Chang Gung Memorial Hospital was obtained from these two participating patients. The recurrent/metastatic tissues were engrafted subcutaneously into NOD/SCID mice.

### PDX study

Local recurrent/metastatic NPC tumor samples were obtained from patients undergoing surgical resection or biopsy. Each sample was immediately cut into small pieces (25–30 mm^3^) in PBS containing 200 U/mL penicillin and 200 μg/mL streptomycin and implanted subcutaneously in the flank region of anesthetized, 4-6-week-old male NOD/SCID mice [32]. In the first round, two mice were each implanted with two pieces of tumor, where tumor volume permitted. Tumor size was measured twice per week with calipers, and the relative tumor volume was calculated. After the xenograft reached ~1 cm^3^ in size, it was excised and sub-implanted into the next passage of mice. The PDX used in these studies were from the first eight passages of each line.

### Microarray analysis of tumor and xenograft

Total RNA was extracted from cells using an RNeasy Mini kit (Qiagen, Valencia, CA) and evaluated by microarray analysis using the Human Whole Genome OneArray v6 (Phalanx Biotech Group, Taiwan), containing 32,679 DNA oligonucleotide probes, each of which is a 60-mer designed in the sense direction. Among the probes, 31,741 corresponded to annotated genes in RefSeq v51 and Ensembl v65 database, and 938 corresponded to control probes. Fluorescent aRNA targets were prepared from 1 μg total RNA samples using a OneArray® Amino Allyl aRNA Amplification Kit (Phalanx Biotech Group, Taiwan) and Cy5 dyes (Amersham Pharmacia, Piscataway, NJ). Fluorescent targets were hybridized to the Human Whole Genome OneArray® in Phalanx hybridization buffer using the Phalanx Hybridization System. After hybridization at 50°C for 16 hours, non-specific binding targets were removed using three different wash steps, and the slides were dried by centrifugation and scanned with an Axon 4000B Scanner (Molecular Devices, Sunnyvale, CA). The Cy5 fiuorescence intensity of each spot was analyzed using GenePix 4.1 software (Molecular Devices).

Signal intensity values for each spot were imported into the Rosetta Resolver System® (Rosetta Biosoftware) for data analysis. The error model of the Rosetta Resolver System, which is able to remove both systematic and random errors from the data, was used to filter out spots for which the flag value was less than 0. Spots that passed all criteria were normalized using the 50% media scaling normalization method. Reproducibility among technical repeat data was tested by calculating the Pearson correlation coefficient (R value >0.975). Normalized spot intensities were transformed to log_2_ ratios of gene expression between control and treatment groups. Spots with a log_2_ ratio ≥1 or ≤ −1 and a *P*-value <0.05 were included in further analyses.

### Drug sensitivity tests in the PDX model

After tumors had been sub-implanted in NOD/SCID mice and xenograft had reached a volume of approximately 150 mm^3^, animals were randomized (3–5 mice with tumors on the right flank per group) and various drugs, including cisplatin, mitomycin-C, fluorouracil, etoposide, gemcitabine, docetaxel, valproic acid, and ganciclovir, were administered via intraperitoneal injection. The following dose schedules were used: cisplatin, 4 mg/kg, 1 time/wk; mitomycin-C, 3 mg/kg, 2 times/wk; fluorouracil, 20 mg/kg, 1 time/wk; etoposide, 12 mg/kg, 3 times/wk; gemcitabine, 2 mg/kg, 5 times/wk; docetaxel, 1 mg/kg, 5 times/wk; valproic acid, 50 mg/kg, 5 times/wk; and ganciclovir, 50 mg/kg, 5 times/wk.

Mice xenografted with the EBV-positive cell line C666-1 served as a control. Final tumor volumes were compared using a two-tailed ANOVA, adjusted for multiple comparisons. A ranked list of effective treatments was provided to the attending physician, who then selected the patient treatment.

### EBV-DNA detection

DNA was extracted from plasma/tissue as described previously [6, 32]. Briefly, 10-mL samples of peripheral blood were collected in EDTA-treated tubes and centrifuged at 1000 × g for 15 minutes. Plasma/tissue DNA was extracted using a QIAamp DNA Blood MiniKit (Qiagen, Valencia, CA). About 500–1000 μL of each sample per column (supplied in the QIAamp kit) was used for DNA extraction. DNA was eluted from each column with 80 μL distilled water [4]. EBV-DNA concentrations were measured by real-time quantitative polymerase chain reaction (PCR) of the *Bam*HI-W region of the EBV genome [32]. Primer and probe sequences, including the dual fluorescence-labeled oligomer, and the detailed procedure have been described previously [6]. The relative EBV-DNA concentration in tissue was expressed as EBV-DNA copies/β-actin gene copies in the same tested samples.

### Detection of EBV-encoded small RNAs

Paraffin-embedded sections from patient tumors or mice xenografts were used for detection of EBER (EBV-encoded small RNAs) by fluorescence *in situ* hybridization. Tissue sections were deparaffinized and pretreated with proteinase K for 10 minutes and then incubated with a fluorescein-conjugated EBER DNA probe (Leica Biosystems, Newcastle, UK) at 37°C for 2 hours. The sections were rinsed in water and incubated with horseradish peroxidase-conjugated anti-fluorescein antibody for 15 minutes before addition of fresh DAB (3,3-diaminobenzidine) substrate to produce an alcohol-insoluble brown intranuclear stain in EBV-positive cells.

## Abbreviations

5-FU: fluorouracil
EBV: Epstein Barr virus
EBER: EBV-encoded small RNAs
CVAT: cytolytic viral activation therapy
GCV: ganciclovir
GEM: gemcitabine
HDAC: histone deacetylase
NOD/SCID: nonobese diabetic/severe combined immunodeficiency
NPC: nasopharyngeal carcinoma
PDX: patient-derived xenograft
SCID: severe combined immunodeficiency
VPA: valproic acid
